# Apprehensions of Morbidly Obese People Regarding Bariatric Surgery

**DOI:** 10.7759/cureus.37098

**Published:** 2023-04-04

**Authors:** Muhammad Zareen, Mutahar Bashir, Shahzeb Khan, Asad Maroof

**Affiliations:** 1 Department of General Surgery, Khyber Medical College, Khyber Teaching Hospital, Peshawar, PAK; 2 Department of Health and Nutrition, Helping Hand for Relief and Development, Islamabad, PAK

**Keywords:** laparoscopic surgery, bariatric surgery, management of obesity, weight-loss, obesity

## Abstract

Introduction

Obesity has emerged as a major public health issue in both developed and developing countries. The prevalence of obesity is on the rise. Bariatric surgery is acknowledged as the most eﬀective and safe solution for this problem. It has been shown to be eﬀective in sustained weight loss and improving quality of life. The aim of this study was to identify the causes of reluctance to have surgery among patients who were potential candidates for weight loss procedures.

Method

Morbidly obese people who were enrolled at Khyber Teaching Hospital, Peshawar, from December 2021 to August 2022 were included in the study. It included hospitalized as well as outpatient appointments. A questionnaire was adopted as the data collection tool.

Result

A total of 107 patients (58 men and 49 women) were enrolled in the study. The median age was 42. Of the 107 patients, 5% (n=5) of the patients were super morbidly obese (BMI >50kg/m2). Seventy-two percent (n=77) of the population considered themselves morbidly obese. Only 22% (n=24) were physically active. Twenty percent (n=21) of the patients reported that they have or are currently trying dietary modiﬁcations for weight loss. Young females were most likely to be on dieting programs. Importantly, 56% (n=60) had never heard of bariatric surgery. Exploring the reasons for reluctance among patients revealed that the concern for surgical mortality was the major hindrance. This was followed by being not interested in committing to surgery and recovery. Concerns regarding cost and ﬁnancing were also the reasons candidates didn't opt for surgical procedures to treat obesity.

Conclusion

The study concluded that there is a serious lack of knowledge and awareness among physicians and the general public regarding bariatric surgery. Most of the patients who were potential candidates for the procedure weren't aware that obesity had a surgical and deﬁnitive treatment. Patients who were aware of the surgical procedure were hesitant to undergo surgery for the management of their weight as they harbored misconceptions, particularly regarding the safety and efficacy of the procedure.

## Introduction

The number of people suffering from obesity has almost doubled since 1980 [1]. The World Health Atlas (2022) predicts that every eighth person in the world will be suffering from obesity by the end of this decade [2]. The numbers are concerning for Pakistan as well, where they hint at an emerging epidemic [3].

Obesity is associated with a number of metabolic, endocrinal, and musculoskeletal disorders [4]. It also has a strong association with hypertension [5]. It causes an increase in cardiovascular complications and puts the obese individual at higher risk of hospitalization from them [6]. Obese individuals have a higher chance of suffering from various cancers owing to a dysregulated metabolism and hormone release [7]. Obesity decreases the quality of life by lending the individual prone to osteoarthritis [8]. It is also a major contributor to disability [9]. Obesity is being held responsible for increasing the risk of death from various diseases and thus reducing the life expectancy of individuals [10].

Obesity management has been dominated by conservative measures for a long time, and lifestyle interventions do result in weight loss, but the benefits are short-lived [11]. Pharmacological management is often compounded by a lack of safe medications with intolerable side effects, even leading to the withdrawal of some from the market [12,13].

Alternatively, surgical management for obesity is gaining ground as its effects on weight loss and, subsequently, on comorbid conditions is unparalleled and outlasts those achieved with conservative treatment [14]. Bariatric surgery confers long-term weight loss and symptom resolution, thus leading to improved quality of life and an increase in life expectancy [15].

Despite this, the number of obese patients undergoing surgery is still underwhelming [16]. This study aims to understand the various reasons for apprehensions regarding surgery as a definitive treatment for obesity.

## Materials and methods

This study is a cross-sectional study. This was conducted at Khyber Teaching Hospital, which is a tertiary care hospital in Peshawar, Pakistan. This study was performed from December 2021 to August 2022. The research proposal was approved by the Institutional Research and Ethical Review Board (IREB).

To obtain demographic details, occupation, physical activity, diet, medical history, and views on bariatric surgery, a questionnaire was used as the data collection tool. The questionnaire was administered by the investigators. Verbal consent was sought from all the participants with the assurance of complete confidentiality and non-disclosure of information. We used consecutive sampling for convenience. A total of 107 people were enrolled. The population includes hospitalized patients as well as outpatient appointments visiting the hospital for other medical issues. The weight and height of patients were measured to calculate BMI. BMI was used to identify and categorize patients. Patients having a body mass index of >35 kg/m2 with associated co-morbidities or a body mass index of >40 kg/m2 without any co-morbidities were defined as morbidly obese and were classified as potential candidates for bariatric surgery [17], and were enrolled for the study. People not falling in this BMI range were not enrolled in the study. Patients who had undergone any weight loss procedure and had opted for surgical methods were excluded from the population. Data was stored and analyzed in SPSS (IBM Inc., Armonk, New York). Measures of frequencies and percentages were documented.

## Results

**Figure 1:**
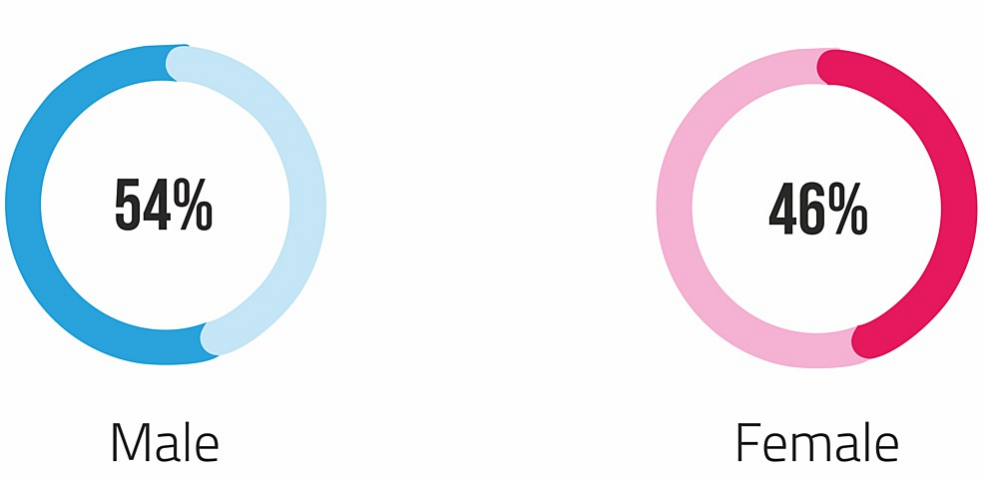
Gender distribution Fifty-eight men and 49 women were enrolled in the study

A total of 107 patients (58 men and 49 women) were enrolled in the study (Figure 1). 

The median age of the population was 42. Five percent (n=5) of the population was super morbidly obese (BMI>50 kg/m2). Eighteen percent were single, 70% were married, and 12% of the patients were either divorced or widowed. Office workers and housewives were the most commonly affected groups. Seventy-two percent (n=77) considered themselves morbidly obese. Table 1 shows the demographic data of all the patients. 

**Table 1:** Demographics

Variable	N
Age	
16 - 25	11
26 - 35	19
36 - 45	30
46 - 55	32
56 - 65	11
66 +	4
Gender	
Male	58
Female	49
BMI	
35 - 39	20
40 - 44	52
45 - 49	30
50 +	5
Occupation	
Office worker	46
Physical labor	7
Student	7
Housewife	38
Unemployed	9

Only 22% (n=24) were physically active, with half of them reporting daily physical activity. Middle-aged men were the most physically active ones. Twenty percent (n=21) of the population reported that they have or are currently trying dietary modifications for weight loss. Young females were more likely to be on dieting programs. Importantly, 56% (n=60) of the potential candidates for weight loss surgery were unaware of the procedure. They had no idea that this disease has a definitive treatment. Forty-four percent (n=47) of the patients who were aware of the surgical procedure were generally hesitant to undergo the procedure (Table 2).

**Table 2:** Weight loss methods tried by patients and their apprehensions towards bariatric surgery

Question	N
Do you consider yourself overweight?	
Yes	77
No	30
Do you exercise?	
Yes	24
No	83
How often do you exercise?	
Once a Week	11
Twice a Week	2
Three or more times a week	11
Have you tried dietary treatment?	
Yes	21
No	86
Are you aware of surgical procedures for weight loss?	
Yes	47
No	60
Why haven't you tried surgical procedures for weight loss?	
Not interested in committing to surgery and recovery	22
Financial reasons	10
Concerned about surgical mortality	25
Unaware that I am a candidate for weight loss surgery	2
Surgery wasn't recommended by my physician	5

**Figure 2:**
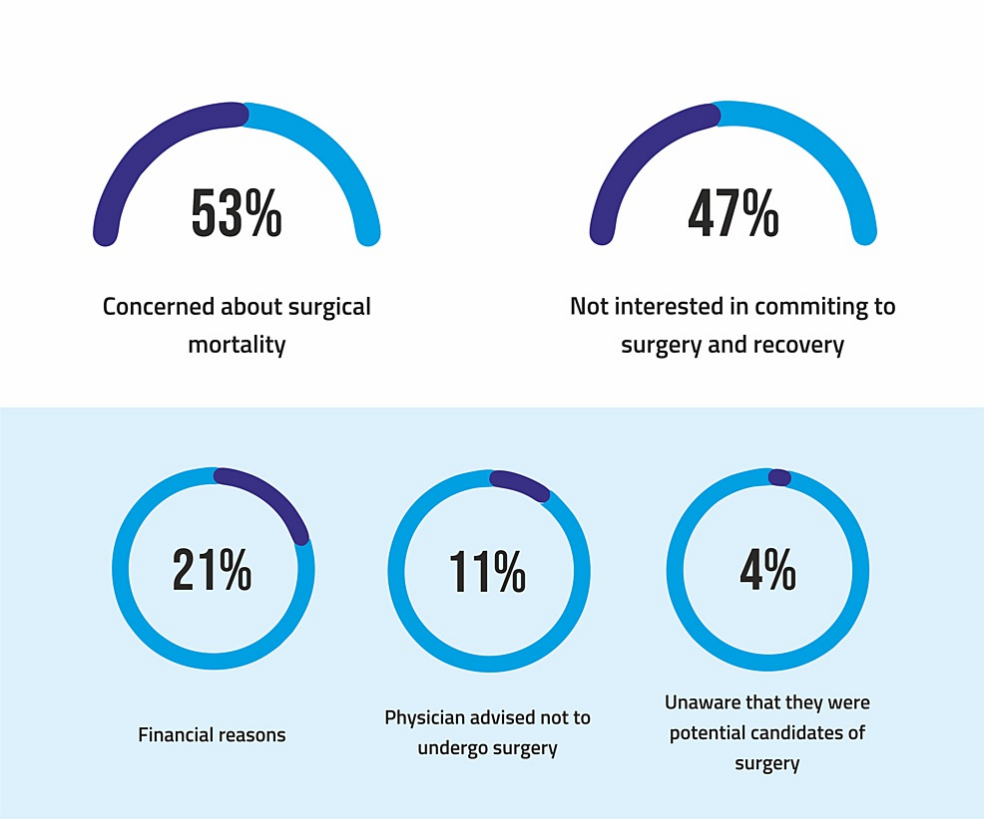
Apprehensions regrading bariatric surgery

The most common reason was the concern regarding the mortality risk. Fifty-three percent (n=25) of the population aware of the surgical procedure shared this concern. Old-age patients (50+) were more concerned, and 47% (n=22) were not interested in committing to surgery and recovery. Middle-aged men were most likely to avoid surgical management because of a lack of commitment. Twenty-one percent of patients didn't opt for surgical options because of financial reasons. Insurance programs were usually not available. Eleven percent of the study population were reluctant as their physicians had advised them against it. Office workers were more likely to consider their physician's recommendation. Finally, 4% (n=5) were unaware that they were potential candidates for a weight loss procedure (Figure 2). 

## Discussion

Obesity has emerged as a concerning public health issue. The sharp rise in numbers has led to the realization of the intricacy of the problem. Keeping the magnitude of the problem in view, management strategies to counter obesity and improvement in them are active areas of research. Lifestyle interventions and pharmacotherapy have been in action for a long time. Recently due to its unparalleled effectiveness in amelioration of symptoms and improving quality of life, bariatric surgery has established itself as the cornerstone of obesity management.

Bariatric surgery has proved to be viable option for effective and sustained weight management [14]. Comparative analysis shows patients managed by surgical options have significant improvement in hypertension, sleep apnea, hyperlipidemia, and coronary artery disease [18]. In obese patients with diabetes, surgery is superior to non-surgical options for remission [19]. To this end, a study showed that 73% of those undergoing surgery achieved diabetes remission as compared to only 13% of those in the non-surgical group [20]. Additionally, it has been found effective in decreasing mortality in the morbidly obese group [15]. However, a significant gap exists between the efficacy of the procedure and its utilization as a management strategy.

Interestingly 56% (n=60) of people who are potential candidates for bariatric surgery have never heard about it. Research shows that obesity is underreported, and interventions are usually not sought in this regard [21]. People usually aren't educated about surgical options by their healthcare physicians. Not hearing about surgical options from your physician has also been reported to challenge the authenticity of the procedure [22].

People aware of bariatric surgery had a lot of reasons for not opting for surgery. The most popular reason was patients being concerned about surgical mortality. Studies show that concern regarding surgical mortality is one of the foremost reasons why obese patients aren't opting for surgical management of their problem [23]. This is a stark contradiction considering the outcomes of surgery. Research has established bariatric surgery to be way safer than the general perception [24]. A recent study showed the mortality rate of bariatric surgery equates to those of routinely performed surgeries such as knee arthroplasty and laparoscopic cholecystectomy [25]. Educating physicians and patients in this regard and equipping them with more information should drastically impact patient views and safety concerns regarding the surgery.

A lot of people were not interested in committing to surgery and recovery. They were concerned about the effectiveness and need of the procedure. Some were of the opinion that this was a cosmetic venture. A study showed that people somehow perceived bariatric surgery as something commercial rather than a treatment modality [22]. This perception is strongly responsible for the underutilization of the procedure. There have been studies that suggest patient awareness about the efficacy of the surgery in long-term sustained weight loss and improvement in comorbidities isn't up to the mark [26].

A number of people reported that financial reasons are the factor that hasn't allowed them to opt for surgical options. A survey of morbidly obese people in Washington revealed that only 29% of the population had coverage for the surgery [27]. The numbers are much more alarming in developing countries. This also has to do with the cosmetic outlook of the procedure.

Few didn't opt for surgery as their physicians had advised them not to go for surgical intervention. A study showed that despite the increasing popularity of the procedure and research evidence establishing its efficacy, physicians have a lot of misconceptions about the procedure [28]. Another research showed that primary care physicians are underestimating the efficacy of surgical management of obesity [29].

Finally, two patients reported that they were not aware that they were potential candidates for surgical procedures. Confident referral of patients by their primary care physicians for bariatric surgery has always been an issue [30].

The present study has some promising findings, but we had a few limitations that might have affected the results and thus could limit the generalization. The geographical limitation was a major one. The present study enrolled patients at Khyber Teaching Hospital, Peshawar. Extending this to different healthcare facilities in future studies would provide us with a better picture of patient's perception regarding the procedures. Secondly, this study was conducted in a hospital, so the sample isn't an exact representation of the population. Increasing the sample size would definitely help. 

## Conclusions

This study established that there is a serious lack of knowledge among physicians and the general public regarding bariatric surgery. Most patients who were potential candidates for the procedure were not aware that the problem had a definitive surgical treatment. Those who knew about the surgical options were hesitant to undergo surgery for the management of their weight. They harbored many misconceptions about the procedure, its outcomes, and its efficacy. Concern for surgical mortality was the primary reason morbidly obese patients did not opt for bariatric surgery. Incognizant physicians further exacerbated the problem. Patients had doubts regarding the results of the procedure and thus were not interested in committing to the surgery and recovery. Work needs to be done to reduce the gap between the efficacy of bariatric surgery and its utilization as a management strategy for obesity.
